# Assessment of a web-based Guided Self-Determination intervention for adults with type 2 diabetes in general practice: a study protocol

**DOI:** 10.1136/bmjopen-2016-013026

**Published:** 2016-12-12

**Authors:** Bjørg Karlsen, Bjørg Oftedal, Silje Stangeland Lie, Berit Rokne, Mark Peyrot, Vibeke Zoffmann, Marit Graue

**Affiliations:** 1 Department of Health Studies, University of Stavanger, Stavanger, Norway; 2 Department of Global Public Health and Primary Care, University of Bergen, Bergen, Norway; 3 Sociology Department, Loyola University Maryland, Baltimore, Maryland, USA; 4 Juliane Marie Centre, University Hospital of Copenhagen, Copenhagen, Denmark; 5 Centre for Evidence-Based Practice, Bergen University College, Bergen, Norway

**Keywords:** DIABETES & ENDOCRINOLOGY, MEDICAL EDUCATION & TRAINING, PRIMARY CARE

## Abstract

**Introduction:**

Self-management is deemed the cornerstone in overall diabetes management. Web-based self-management interventions have potential to support adults with type 2 diabetes (T2DM) in managing their disease. Owing to somewhat ambiguous results of such interventions, interventions should be theory-based and incorporate well-defined counselling methods and techniques for behavioural change. This study is designed to assess the effectiveness of a theory-driven web-based Guided Self-Determination (GSD) intervention among adults with T2DM in general practice to improve diabetes self-management behaviours and glycosylated haemoglobin (HbA1c).

**Methods and analysis:**

A complex intervention design based on the framework of the UK Medical Research Council is employed as a guide for developing the intervention, assessing its feasibility and evaluating its effectiveness. The study consists of three phases: (1) the modelling phase adapting the original GSD programme for adults with T2DM, using a qualitative design, (2) feasibility assessment of the adapted intervention on the web, employing qualitative and quantitative methods and (3) evaluating the effectiveness of the intervention on diabetes self-management behaviours and HbA1c, using a quasi-experimental design. The first phase, which is completed, and the second phase, which is underway, will provide important information about the development of the intervention and its acceptability, whereas the third phase will assess the effectiveness of this systematically developed intervention.

**Ethics and dissemination:**

The Norwegian Regional Committee for Medical and Health Research Ethics (REK west number 2015/60) has approved the study design. Patients recruited in the different phases will fill out an informed consent form prior to inclusion and will be guaranteed anonymity and the right to withdraw from the study at any time. The results of the study will be published in peer-reviewed journals, electronically and in print, and presented at research conferences.

**Trial registration number:**

NCT02575599.

**Strengths and limitations of this study**

- Use of a complex intervention design.
- Developing a theory-based Guided Self-Determination programme for adults with type 2 diabetes (T2DM).
- Testing a web-based intervention in general practice in order to increase self-management among adults with T2DM.
- The assignment of patients to the intervention and control groups will not be random.
- The quasi-experimental design requires cautiousness about drawing inferences and conclusions from the data.

## Introduction

Type 2 diabetes (T2DM) is a chronic disease and a growing public health problem worldwide. Its prevalence is increasing rapidly in many countries, including Norway.^1^ In Norway, general practitioners (GPs) and registered nurses working with GPs have traditionally been responsible for the care of T2DM. In addition to annual check-ups and regular consultations (3–4 times a year) with GPs, most people with T2DM are offered a structured educational programme at the hospital on diagnosis to learn how to manage their disease.^2^ Yet, research indicates that only one of eight patients with T2DM achieves the recommended treatment goals of glycaemic control, cholesterol and blood pressure.^3^ Consequently, there is ample room to improve diabetes care.

At present, there is no cure for T2DM. Self-management of the disease is, therefore, a key factor in achieving adequate blood glucose control and preventing long-term complications.^4^ Besides, it is deemed the cornerstone of overall diabetes management.^5^ ^6^ Diabetes self-management is described as an active, proactive and ongoing process that includes behaviours such as healthy eating, regular physical activity, blood glucose monitoring and medication taking, as well as problem-solving and healthy coping.^4^ Achieving appropriate self-management is, however, complex.^5^ ^7^ Owing to this complexity, research has indicated that individuals consider attainment of treatment goals challenging, and many individuals fail to achieve optimal treatment outcomes.^8^ Given the fact that adults with T2DM implement the majority of self-management tasks,^9^ these tasks need to be facilitated by support from healthcare professionals.^10^ Such support is required for implementing and sustaining coping skills and behaviours needed to self-manage on a continuing basis.^11^ However, a Norwegian study has suggested that support from healthcare practitioners has a weak influence on self-management behaviours.^12^

To promote daily self-management for adults with T2DM, several educational and behavioural programmes have been developed. Moreover, national standards for diabetes self-management education (DSME) and support have been designed to serve as a guide for diabetes educators.^13^ Yet, reviews have demonstrated moderate effects of a number of previous interventions to improve self-management.^7^ ^14^ However, a recent review shows that it is important to include support components to best train people in their self-care.^15^ Thus, ongoing educational, behavioural and clinical support is needed following DSME to sustain changes made during DSME.

The internet has increasingly been used for delivering interventions designed to promote self-management and health behaviour change.^16–18^ Reviews investigating a number of web-based interventions for promoting self-management of T2DM have demonstrated substantial improvements in health behaviours such as self-monitoring, physical activity and diet, as well as health-related outcomes such as weight, glycaemic control and emotional distress,^16^ ^17^ ^19^ whereas other studies have demonstrated moderate effects.^19^ ^20^ Owing to these somewhat ambiguous results, it has been suggested that future internet interventions should be theory-based and incorporate counselling methods and techniques for behavioural change.^17–19^ Research has also demonstrated that internet self-management support without tailored feedback has been associated with greater dropout than when provided in combination with tailored feedback.^21^ In addition, previous research has suggested that diabetes self-management support for adults with T2DM is effective when delivered in a community context.^5^ Furthermore, web-based interventions in community-based general practice seem to be convenient, easily accessible and less costly,^22^ and may increase interest and participation in diabetes management that are needed in adults with T2DM.^23^

There is still potential for the development of effective and reliable web-based self-management interventions. In this study protocol, we respond to these challenges by describing the development of a web-based Guided Self-Determination (GSD) programme for adults with T2DM in general practice. The GSD programme has a theoretical foundation in Self-Determination Theory^24^ as well as empowerment^25^ and life-skills theories.^26^ It is designed to be a self-management support programme and has been effective in individual and group training of adults with type 1 diabetes (T1DM).^27^ Moreover, research has demonstrated that application of GSD for people with T1DM in a clinical context has been approved by patients. It is also suggested that GSD can be adapted to other contexts with other patient groups.^28^ ^29^ Since T2DM is different from T1DM regarding pathophysiology, symptoms, treatment and diabetes self-management, it is timely to consider how a web-based GSD intervention for adults with T2DM might improve self-management behaviours and subsequent healthcare outcomes in a general practice. To the best of our knowledge, this approach has not yet been investigated.

In this study, we present a study protocol for (1) developing a web-based GSD programme adjusted for adults with T2DM, (2) assessing its feasibility and (3) evaluating its efficacy in general practice.

### Aim

The overall aim of this study is to assess the effectiveness of a web-based GSD programme among adults with T2DM in general practice in order to improve diabetes self-management and glycosylated haemoglobin (HbA1c) through enhanced patient activation, self-management competence and autonomy.

## Methods and analysis

### Study design

We use the framework of complex interventions proposed by the UK Medical Research Council (MRC)^30^ ^31^ as a guide for developing the intervention, assessing its feasibility and evaluating its effectiveness. This framework is recommended for the development of interventions containing several interacting components.^30^ The development of the web-based intervention is informed by literature reviews of existing web-based interventions^16^ ^17^ ^19^ and previous experience in developing, testing and evaluating the GSD among adults with T1DM.^27^

### Study overview

The study detailed in this protocol consists of three phases covering the first three stages of the MRC framework:^30^ (1) the modelling study, which has already been completed, (2) the feasibility study, which started in spring 2016 and is underway and (3) the evaluation study, which is planned to start in 2017. An overview of the different phases containing aims, methods and participants, respectively, is depicted in table 1.

**Table 1:** Study overview

Phases	Aims	Methods	Participants
Phase 1. The modelling study	*Step 1*: Identifying areas to be changed from the original GSD. *Step 2*: Adapting the GSD to patients with T2DM, and for internet delivery.	Qualitative approaches with individual interviews in both steps, using qualitative content analysis. A resource group (12 in total) together with a group of user representatives (5 in total) identified adjustments applicable to adults with T2DM and for internet delivery.	*Step 1*: Eight patients with T2DM recruited from GPs. *Step 2*: Eight patients with T2DM recruited from GPs.
Phase 2. The feasibility study	Pretesting the adapted GSD intervention on the web and assessing its feasibility.	Quantitative: Pre–post design. Qualitative: Individual interviews at the end of the programme, using qualitative content analysis.	40 patients with T2DM who have participated in the web-based GSD adapted for T2DM will be recruited from GPs.
Phase 3. The evaluation study	Evaluating the effectiveness of a structured theory-driven web-based GSD intervention on self-management behaviours and HbA1c.	Quasi-experimental design with outcome assessments at baseline, end of programme and 6-month postprogramme.	A total of 172 patients with T2DM will be recruited from GPs (intervention group n=86, control group n=86).

GP, general practitioners; GSD, Guided Self-Determination; HbA1c, glycosylated haemoglobin; T2DM, type 2 diabetes.

### Phase 1: the modelling study

#### Training the nurses to deliver GSD

We invited four nurses working in different general practices and with extensive experience in counselling patients with T2DM to participate in a structured and supervised training programme to develop their counselling skills in nurse-led consultations. Three of the nurses had formal postgraduate education in diabetes care and the fourth had long experience in diabetes care. The training programme included a 4-day course in the original GSD approach developed for people with T1DM over a period of 9 months. It addressed theoretical and practical topics and employed the method in face-to-face consultations with adults with T2DM. The training also included use of several structured reflection sheets. The development of these sheets was inspired by Arborelius and Bremberg^32^ and based on grounded and selected formal theories.^33–35^ The nurses were trained in three advanced professional communication skills: mirroring, active listening and values clarification response.

#### Description of the original GSD intervention

The original GSD programme consisting of 7 face-to-face consultations with 21 structured reflection sheets was designed to guide patients and professionals through mutual reflections illustrated on semistructured worksheets. Reflections recorded on worksheets are intended to empower the patient to become self-determined, with adequate life skills to manage challenges in diabetes self-management. An overall challenge or problem was reflected on in a central sheet called dynamic judgement building inspired by Bos.^34^ The reflection sheets encompass four themes about the patient–provider relationship, life with diabetes, the relationship between ideal and reality and change work. It is a six-stage process, including (1) the establishment of a mutual relationship with clear I–you-borders, (2) self-exploration, (3) self-understanding, (4) shared decision-making, (5) action and (6) feedback from action. Prior to the consultations, the worksheets are introduced with the purpose of stimulating the patient's reflection processes between the consultations and of preparing for the next consultation.^27^ ^36^ ^37^

#### Adjusting the GSD programme to patients with T2DM in two steps

The intention of the modelling study was to guide the adjustment of the GSD intervention for web-based delivery to patients with T2DM.^30^ The phase consisted of two steps (table 1). At the initial step, it was essential to identify areas that needed to be changed from the original GSD intervention. As part of the training programme noted above, each of the nurses recruited two patients with T2DM from their general practices to participate in seven face-to-face consultations using the semistructured reflection sheets. The inclusion criteria were: (1) diagnosed with T2DM, (2) adults aged ≥18 years, (3) disease duration >3 months and (4) ability to communicate in Norwegian. After participating in the seven face-to-face consultations, patients were interviewed individually about their experiences with the GSD approach, how it worked for them and potential suggestions on how to tailor-make the approach more appropriate for T2DM. The findings of these interviews were analysed using qualitative content analysis.^38^ A resource group (12 in total) was established with 5 researchers, 3 nurses experienced in using the GSD for people with T1DM and 4 nurses carrying out the intervention (table 1). In addition, to provide patients' perspectives on self-management support, a five-person group of user representatives—two people with T2DM selected from the Norwegian Diabetes Association, two nurses and one GP experienced with working with adults with T2DM—was involved in the discussions. Analyses from the discussions were based on findings from the interviews, experiences from the nurses conducting the programme and perspectives of the users about the relevance of the suggested adjustments. Through this process, the number of face-to-face consultations was reduced from seven to four to make it more time efficient. In addition, the number of worksheets to be used at each consultation was reduced. Since it was important to maintain the essential elements of the GSD, we included the central work sheets on dynamic judgment of current and future problem solving. Table 2 illustrates the number and the content of the reflection sheets used in the intervention for T2DM.

**Table 2:** The GSD adapted to T2DM

The first session	Preparing for subsequent consultations: Invitation to work together The HbA1c measurement
Consultations:	Reflection sheets (RS)
Your life with diabetes	RS 1a. Important events and periods in your life RS 1b. At present, what do you find difficult about living with diabetes? RS 1c. Unfinished sentences—your needs, values, habits and opportunities RS 1d. A picture, metaphor or expression of your life with diabetes
Focus for change	RS 2a. Room for diabetes in your life RS 2b. Your plans for changing your way of life
Work with changes	RS 3a. Clarification of challenge in your life with diabetes RS 3b. Previous problem-solving: thoughts, feelings, goals and actions RS 3c. Dynamic problem-solving
Changes in daily life	RS 4a. Blood sugar checks and your reasons for checking RS 4b. New strategies and long-term plan for change RS 4c. Dynamic judgement of current and future problem solving RS 4d. ‘Pros and cons’

GSD, Guided Self-Determination; HbA1c, glycosylated haemoglobin; T2DM, type 2 diabetes.

In the second step, the same nurses each recruited two additional patients with T2DM from their general practices according to the same inclusion criteria mentioned above. After participating in the four face-to-face GSD consultations adapted for T2DM, patients were interviewed individually about their experiences with the adapted approach. Preliminary findings from the interviews indicated that the patients were satisfied with the adapted version. The aforementioned resource group, together with the group of user representatives, did not recommend further adjustments.

### Phase 2: the feasibility study

#### The adapted GSD intervention on the web

The intervention is delivered through the platform MinJournal (‘My Chart’), developed at the Oslo University Hospital, Norway. It allows secure online communication between patients and healthcare professionals. The portal demands electronic identification by using BankID to reach the necessary security level 4, which is required when transferring sensitive data and medical journal information in the Norwegian healthcare system.

The worksheets applicable for T2DM are included in the web pages (see table 2). The intervention will consist of four e-consultations over 12–16 weeks. Information about the reflection sheets, together with information on how to communicate via the web portal, will be presented face to face at the first appointment with the nurse. Through the existing web-based platform, the subsequent four e-consultations will allow for communication between patients and nurses. The platform allows the participants to fill in the reflection sheets using their own words and drawings to express and reflect on their experiences and difficulties with diabetes management in daily life as well as to formulate behavioural goals and plans to achieve improved self-management. It also permits feedback from the nurses on these reflections, goals and plans via secure emails. Each person will be able to access the web-based programme from his or her own computers or other electronic devices.

#### Participants

In addition to the four nurses participating in the modelling phase, four new GSD-trained nurses will pilot the feasibility study (n=8). One of these four had formal postgraduate education in diabetes care, whereas the others had long experience in working with adults with T2DM. To obtain a varied picture of how the participants perceive the GSD programme, each nurse will recruit five patients with T2DM (n=40) from their general practices to participate in the web-based intervention. In addition to the inclusion criteria used in the modelling phase, participants are required to have access to the internet and Bank ID. Patients with cognitive impairment and/or severe comorbidity that would interfere with participation in the intervention will be excluded from the study.

#### Data collection and outcomes

This phase will employ quantitative and qualitative assessments of the feasibility of the recruitment strategies, programme acceptability and satisfaction, as well as evaluation of the measures to be used in the evaluation study (table 1). The elapsed time to fill out the questionnaires, accuracy of biographic and clinical variables and handling of the format will especially be assessed. The qualitative approach includes individual interviews with patients at the end of the intervention. On the basis of findings from the questionnaires and the interviews, we will determine features of the intervention components to be valuable and those in need of further development. This information, together with recommendations from the expert group, will be used to finalise the intervention for use in the third phase of the study (see table 1).

#### The quantitative approach

Data will be collected at baseline and at the end of the intervention using self-reported questionnaires.

##### Primary outcomes

Self-reported goal attainment questionnaire. The questionnaire developed by the research team will assess to what degree the patients have achieved diabetes goals with the following two items: (1) What were your change goals? and (2) To what degree have you achieved your goals?

The 13-item Patient Activation Measure (PAM). PAM developed by Hibbard *et al*^39^ ^40^ will assess patient knowledge, skills and confidence in self-management of the patient's health and chronic conditions. The total score ranges from 0 to 100 (best).^41^ PAM has been validated in a previous Norwegian study.^42^

##### Secondary outcomes

The Health Care Climate Questionnaire (HCCQ), a six-item scale based on the original 15-item HCCQ,^43^ ^44^ will measure the patients' perceptions of the degree to which they perceive autonomy support from healthcare providers.^43^ ^45^ Each item is scored on a seven-point scale ranging from ‘strongly disagree’ to ‘strongly agree’. Higher scores indicate greater autonomy support from healthcare practitioners.

The degree of competence perceived by patients in managing diabetes will be assessed by using the four-item Perceived Competence for Diabetes (PCD) scale.^44^ ^46^ The items have a seven-point Likert scoring format ranging from ‘not at all true’ to ‘very true’. Higher scores indicate a better perceived competence.

The 21-item Treatment Self-Regulation (TSRQ) scale developed by Ryan and Connell^47^ will measure the degree to which a person's motivation for a particular behaviour or set of behaviours is relatively autonomous, controlled or amotivated. The scale has been modified and adapted to assess various health behaviours.^48^

The 20-item Diabetes Management Self-Efficacy Scale (SE-T2DM) developed by Bijl *et al*^49^ will assess diet, feet control, medical treatment/control and exercise ability expectations. The respondents are asked to indicate their ability expectations on a five-point Likert scale ranging from ‘no, definitely not’ to ‘yes, definitely’. Higher scores represent greater expectations of the ability to perform necessary self-management activities.

The 14-item Summary of Diabetes Self-Care Activities (SDSCA)^50^ will assess self-care activities of people with diabetes such as general diet, specific diet, exercise, blood glucose monitoring, foot care and smoking. The instrument has extensively been used in diabetes research and has shown satisfactory reliability and validity.^50^ The respondents are asked about the frequency of performing the different self-care activities over the preceding 7 days. Scores range from 0 to 7 days; higher scores indicate greater frequency of performing self-care activities.

Emotional well-being will be assessed by using the WHO 5-item Well-Being Index (WHO-5).^51^ ^52^ It is conceptualised as a unidimensional concept that contains five positively worded items. The degree to which these positive feelings were present in the past 2 weeks is scored on a six-point Likert scale ranging from 0 (not present) to 5 (constantly present).

Diabetes-specific distress will be assessed using the 17-item version of the Diabetes Distress Scale (DDS).^53^ The DDS has four subscales: emotional burden (five items), physician-related distress (four items), regimen distress (five items) and diabetes-related interpersonal distress (three items). The responses are on a scale (1–6) from ‘not a problem’ to ‘a very serious problem’. The scale has been validated in a previous Norwegian study.^54^

In addition, clinical parameter, such as HbA1c, will be measured by a blood sample at the GP office at baseline and at the end of the intervention. Self-reported height and weight (for body mass index calculating) and medical treatment will be collected as well. The accessibility and practice of the web-based portal will be measured by asking participants to write down the number of log-ons, time spent on filling the reflection sheets, time spent on e-consultations and the number of feedbacks from the nurses.

#### The qualitative approach

The intervention process, such as facilitators and barriers to the adoption of the web-based GSD, including the quality of the communication via emails with the nurses, will be studied. All participants in the GSD intervention will be invited to take part in individual interviews. The interviews will use a semistructured interview guide developed by the research team, and they will be digitally audio-recorded and transcribed verbatim.

#### Data analysis

Quantitative data will be analysed using SPSS V.22 (IBM Corp, Armonk, New York, USA). Descriptive statistics will include frequencies, mean value, SD and CIs. Qualitative content analysis^38^ will be used to analyse the data from the interviews.

### Phase 3: the evaluation study

#### Quasi-experimental design

The effectiveness of this phase will be evaluated in a quasi-experimental design with one intervention and one control group. We hypothesise that a web-based GSD intervention will result in improvements in self-management behaviours and HbA1c compared with regular care in general practice.

#### Recruitment and data collection

The study will be carried out in general practices in the western part of Norway. To accomplish the requirement of trained GSD nurses, trained nurses working in general practices will conduct the GSD intervention. Participants with T2DM to be assigned to the intervention group will therefore be recruited from the practices where the nurses are working. Participants assigned to the control group will be recruited from other general practices with employed registered nurses without the GSD training. They will continue with their regular consultations in general practice. Inclusion and exclusion criteria are the same as described for the feasibility study.

All patients considered eligible for the study will be invited by mail to participate. Those who agree to participate and return a written informed consent will be enrolled and the nurse at each selected general practice will then make an appointment with the patient to start the GSD intervention.

#### The intervention

Based on findings from the feasibility study, refinements of the web-based intervention will be made via ongoing discussions with the research team, user representatives and diabetes nurses before deciding on the final application to the web. We intend to retain the essentials of the GSD approach and the selected worksheets addressing central GSD issues. In addition, we plan to base our intervention on the five consultations with the nurse assessed in the feasibility phase; the first appointment face to face, then the subsequent four e-consultations with selected structured worksheets aiming to improve diabetes self-management and HbA1c through patient activation, enhanced self-management competence and autonomy.

#### Outcome assessments

Measurements are scheduled at three time points: at baseline, 12–16 weeks later at the end of the intervention and 6 months after finishing the intervention.

The patient-reported outcome measures to be collected during this evaluation study will be finalised during the feasibility study, but are likely to be the same as described in the feasibility study. Regarding the goal attainment measure, we expect that the intervention group will report having more goals, more ambitious goals and more progress towards attaining their goals. Other hypothesised benefits of the intervention include increases in PAM scores on patient activation, HCCQ scores on autonomy support, PCD scores on competence, TSRQ scores on autonomy and control, SE-type 2 scores on self-efficacy, SDSCA scores on self-care activities, WHO-5 scores on emotional well-being and decrease in DDS scores on diabetes distress. Clinical outcomes described in the feasibility study (ie, HbA1c) will also be collected at all three time points. We predict reductions in these outcomes relative to the control group.

#### Data analysis

The quantitative data will be analysed using SPSS V.22 (IBM Corp, Armonk, New York, USA). Descriptive statistical analyses will include frequencies, means, SDs and CIs. The effect of the intervention on outcomes will be analysed by regression analyses with the following covariates: baseline scores, age and gender.

A supplementary analysis will assess whether patient-determined goals predict change on outcomes, that is, whether patients' change goals for particular self-care activities or psychological outcomes predict greater improvements on those outcomes (eg, for analysis of frequency of blood glucose self-monitoring, we will assess the potential impact of each patient's goal, if any, for blood glucose self-monitoring). We will also explore the possibility of pooling outcome results according to whether a patient had a goal to improve that outcome, for example, estimating the pooled effect size for change in each patient's primary chosen goal.

Additional analyses will assess patient usage of and satisfaction with the intervention tools, as well as facilitators and barriers to adoption of the web-based GSD, including the quality of the communication via secure messages with the nurses. We will also examine the nurses' perceived facilitators and barriers to the adoption of the web-based GSD.

#### Sample size calculation

In GSD-like interventions, identification of a primary outcome differs from a traditional intervention study in that each individual participant (rather than the study director) determines the goals of the intervention. In essence, different patients have different primary outcomes. Nevertheless, for purposes of calculating power, a primary outcome must be identified. Since GSD is designed to increase patient involvement in goal selection and behaviour change, we have selected patient activation as the primary outcome. Therefore, change in patient activation is the primary end point as measured by the PAM scale total score. According to Steinsbekk,^55^ a detected difference in PAM score of six points (from 66.4 to 72.4) (SD 11.1) with 90% power and 5% significance level, the sample size must be 72 in each group. Given an expected dropout rate of 20%, we will include at least 172 patients in our study, 86 patients in each group.

## Dissemination

All patients recruited in each phase will fill out an informed consent form prior to inclusion and will be guaranteed anonymity and the right to withdraw from the study at any time. The results of the study will be presented at research conferences and published in peer-reviewed journals, electronically and in print.

## Discussion

In this study protocol, we have described a complex intervention design for modelling, assessing feasibility and evaluating a GSD intervention. While the first two phases of this study will provide information on designing the intervention and its acceptability, the third phase will assess the effect of this systematically developed intervention on health outcomes such as patient activation, psychological outcomes, self-management behaviours and clinical outcomes. Moreover, we will identify factors that relate to the website use and its effectiveness in order to guide future web-based intervention development and implementation. We will gain insights into the participants' experiences of using the web-based programme at home, in their own time and hopefully increasing flexibility in everyday life.

### Strengths and limitations

The theory-driven and evidence-based approach to develop the web-based GSD intervention for adults with T2DM in general practice will constitute the backbone of this study and a novel contribution. Potential strengths would be the systematic refinement of the intervention based on data from the modelling and the feasibility studies, our use of qualitative and quantitative approaches to assess the feasibility and the quasi-experimental design to test the effectiveness of the intervention. These phases in the research process as outlined in the MRC framework of complex interventions are fundamental to create high-quality interventions and assess their effectiveness in everyday practice. Conceivably, the inclusion of patients, nurses and GPs throughout the project phases will have widened the range of user views and perspectives applicable in the intervention improvement process.

Conducting the web-based GSD intervention in routine general practices for patients with T2DM adds to the external validity of the study. Nurses working in general practices and trained in GSD can use the intervention as support for their standard consultations, which may result in improved diabetes care. Besides, GPs and nurses will play an active role in shaping the study intervention. Arguably, through ongoing deliberations about findings and modifications of the GSD between the researchers, nurses, GPs and user representatives, the validity of the study will be improved.

Possible limitations, especially those related to our evaluation study in the third phase, include the fact that assignment of patients to the intervention and control groups will not be random. This sampling, therefore, requires cautiousness about drawing inferences and conclusions from the data. In addition, the power of the study to assess improvement on any single outcome is reduced by the fact that only a subset of patients will have any particular outcome as a goal of their participation. In effect, the evaluation study can be thought of as a set of substudies, one for each outcome. However, our analyses will be designed to compensate for this departure from the top-down, researcher-driven design used in traditional non-personalised interventions. Finally, long-term (ie, > 6 months) effects of the web-based GSD intervention will not be assessed in this study. However, if this intervention is judged effective in the current study, this should provide the information necessary to conduct a definitive study of its long-term effects.

To the best of our knowledge, this will be the first study examining the GSD intervention adapted to a web-based version for adults with T2DM in general practice. We therefore expect that the results of this study will add significantly to the body of knowledge regarding web-based interventions for improving self-management among people with T2DM.
